# Factors associated with Posttraumatic Stress Disorder and Its Coping Styles in Parents of Preterm and Full-Term Infants

**DOI:** 10.5539/gjhs.v6n3p65

**Published:** 2014-02-20

**Authors:** Maryam Ghorbani, Mahrokh Dolatian, Jamal Shams, Hamid Alavi-Majd, Samira Tavakolian

**Affiliations:** 1 Shahid Beheshti University of Medical Sciences, Tehran, Iran; 2 Department of Midwifery, Shahid Beheshti University of Medical Sciences, Tehran, Iran; 3 Department of Psychiatry, Behavioral Research Center, Shahid Beheshti University of Medical Sciences, Tehran, Iran; 4 Department of Biostatistics, School of Paramedical Sciences, Shahid Beheshti University of Medical Sciences, Tehran, Iran

**Keywords:** prematurity, parents, posttraumatic stress, coping styles with stress

## Abstract

**Introduction:**

Birth of a premature infant and subsequent neonatal intensive care leads to psychological distress and trauma in parents. A large proportion of mothers show signs of trauma long after discharge from hospital. Fathers of premature infants are known to experience more stress than fathers of full-term infants. The sorrow experienced by parents of preterm infants is significantly higher than that experienced by parents of full-term infants because they have not been adequately prepared for the experience of birth, and need to cope with the stress caused by the clinical state and intensive care of the infant.

**Method:**

This was a descriptive-comparative study conducted in medical centers of Qom, Iran in 2012. In this study, 82 couples (164 mothers and fathers), participated in two groups as parents of preterm and full-term infants and completed demographic, midwifery, posttraumatic stress disorder, Spielberg anxiety questionnaires, and the Coping Inventory with Stressful Situation within 2 months after birth of their infant. Data were analyzed using Chi-square, Fisher’s exact, Mann-Whitney, independent t tests, logistic regression, and Repeated measures ANOVA in SPSS-18 software.

**Results:**

Posttraumatic stress disorder in preterm group mothers was significantly higher than in term group mothers (P=0.03), but no significant difference in this disorder was observed between fathers in these groups. There was a significant difference in coping styles with stress between mothers in the two groups (P<0.001) and between fathers in the two groups (P<0.001). Logistic model showed a significant correlation between posttraumatic stress and housing and coping strategies with stress in mothers.

**Conclusion:**

Parents of premature infants are more exposed to psychological disorders, and there is a need to adopt educational approaches to improve parents’ coping ability with preterm infant’s circumstance.

## 1. Introduction:

With prevalence of 5 to 13%, preterm birth is referred to a birth that occurs between the 20^th^ and 37^th^ gestational week (Subramaniam et al., 2012, Jahromi et al., 2011, Lima de Souza and Pinheiro-Fernandes, 2010). Outcomes of preterm infants are associated with such factors as infant’s status, parents’ attitude, socio-economic parameters, infant’s characteristics, mother-infant relationship, and the family atmosphere (Pierrehumbert et al., 2003). Parents of premature infants are more exposed to stress associated with physical appearance, infant’s condition, and parenting problems (Melnyk et al., 2006). Birth of a premature infant and subsequent neonatal intensive care lead to psychological distress and trauma in parents. A large number of mothers show signs of trauma long after infant’s discharge from hospital, and express painful memories 6 to 18 months after. These memories are often undesirable and intrusive, and recalling them is usually associated with attempts to avoid remembering prematurity of the infant. Intrusiveness, avoidance, and hyperarousal are the three signs of posttraumatic stress disorder (Jotzo & Poets, 2005). It seems the degree to which a mother can alleviate feelings of loss and grief associated with premature delivery can affect mother-child relationship (Shah et al., 2011).

It has even been observed that fathers of preterm infants experience more stress than fathers of full-term infants. Stressful factors in the transition to fathering role involve changes in fathers’ role and absence of the mothers due to their involvement in the care (Foa & Tolin, 2000). Still, there is little information about fathers’ experience, as mothers are normally more attended to (Melnyk et al., 2006). Emotions of fear, anxiety, low self-esteem, failure, and inability to cope with stress have been described in parents’ care for the premature infant. The grief experienced by parents of preterm infants is significantly higher than that in parents of full-term infants because they are not prepared for the experience of birth, and there is a need for parents to cope with the stress caused by clinical condition and intensive medical care. Following assessment of a stressful situation, a person begins to adopt different coping strategies for the event (Jotzo & Poets, 2005).

Therefore, given the importance of early diagnosis of prematurity, posttraumatic stress disorder and associated factors, interventions to increase preterm infants’ parents’ ability to cope, and neglecting fathers in most studies, the researcher decided to compare posttraumatic stress disorder and coping strategies in parents of term and preterm infants.

## 2. Patients and Methods

This descriptive-comparative study was conducted on all parents of premature and mature infants who had attended health centers in Qom to receive postnatal care from December to March 2012. Inclusion criteria included: no mental illness, no history of any mental disorders, no mental medications such as antidepressants, or psychotropic substances, and no neurological or congenital defects in newborns. Based on statistics, there were 82 couples in each group. Multistage sampling was used in the present study. Qom city was first divided into four economic classes (the first stage of sampling was cluster sampling). Then all health centers of Qom city were listed and the entire sample was distributed based on existing data and in proportion to the volume of patients (the second stage of sampling was quota sampling). In the next step, centers were randomly selected within each region for the sampling. This study was approved in 20 October 2012 in the Deputy for Research of Shahid Beheshti University of Medical Sciences. To comply with principles of ethics, the following was carried out by the researcher: First, the researcher obtained permission letter from officials at Shahid Beheshti University of Medical Sciences and presented the letter to Qom University of Medical Sciences. During sampling, participation in the study was voluntary, and subjects could decline to continue at any stage in the study. The researcher first introduced herself to participants and explained objectives of the study to them. Participants were assured of confidentiality of information. After examination of data, parents in need of counseling, or treatment were referred to a psychologist or a psychiatrist. Data were analyzed using Chi-square, Fisher’s exact, Mann-Whitney, independent t tests, logistic regression, and Repeated measures ANOVA in SPSS-18 software.

**The questionnaires for this study include**

**Section I**

Questionnaire regarding father’s information contains 2 parts. The first part includes questions about demographic characteristics, history of psychiatric illness in first-degree relatives (6 questions). The second part contains information about the unpleasant events which the father may have experienced during his spouse’s pregnancy (27 questions).

**Questionnaire regarding mother’s information** consists of 4 parts.

**Part I**

The first part includes questions about demographic characteristics, socioeconomic status of the family including housing, family income, and family size (12 items). The second part contains information about the mother’s pregnancy including questions about: previous pregnancies, methods of contraception, prenatal care, the rate of satisfaction with midwife’s care during childbirth, diseases experienced during pregnancy, insurance type, delivery type, and hospitalization after delivery (38 questions). The third part includes neonatal factors including questions about the baby’s gender, type of pregnancy, baby’s disease(s) after birth, infant’s history of admission to hospital, parent’s satisfaction with the baby’s gender, baby’s gestational age at birth, baby’s weight at birth, baby’s nutrition, problems in taking care of the newborn, ability to pay baby’s hospitalization costs (15 questions). The fourth part includes questions related to unpleasant events in pregnancy (27 questions).

In this study, content validity was used to determine the validity of demographic and obstetric questionnaire. To determine reliability of this questionnaire, test retest was used. The reliability coefficient was calculated as 98 percent.

**Part II**

**Post-traumatic stress disorder symptoms scale:** To diagnose post-traumatic stress disorder (PTSD), the severity of symptoms was evaluated based on DSMIV (Diagnostic and Statistical Manual of Mental Disorders); which contains 17 questions with Likert scale. Each question contains a short question and the person’s answers are graded from zero (not at all) to 3 (5 or more times a week). In this scale, the frequency and severity of symptoms have been incorporated. The reason is that some PTSD symptoms can be evaluated based on frequency of its occurrences (such as trauma-related nightmares) and others can be described based on their severity (hyper arousal). Four questions related to re-experiencing, 7 questions related to avoidance, and 6 questions related to motivational response. In case of having one or more symptoms related to re-experiencing, 3 or more symptoms of avoidance, 2 or more symptoms related to motivational reactions, PTSD was diagnosed (Foa and Tolin, 2000; Shaban et al., 2013).

In Iran, Mirzamani et al. assessed validity of this scale using concurrent validity method in 2006 (r = 0.79, P<0.001). Its reliability in test-retest was 74% in various studies and 88% in Cronbach’s alpha method (Mirzamani et al., 2006)

**Part III**

**Coping Inventory with Stressful Situation (CISS):** This questionnaire is used to assess coping styles of people in stressful situations. This test includes 48 items, with 16 items for each of the coping dimensions of problem-oriented, emotion-oriented, and anxiety. Scoring is based on the 5-point Likert scale, from never (1) to very much (5). Shokri et al. in their 2008 study found internal consistency of this questionnaire 0.55-0.64 and its reliability with Cronbach’s alpha 0.7-0.86 (Shokri et al., 2008)

## 3. Results

According to table 1, mean age of mothers in the full-term group was 28.2±4.5 years and in the preterm group 27.6±6.2, and mean age of fathers in the full-term group was 32.8±4.8 and in the preterm group 31.7±6.2 years. 51.2% of term group mothers and 43.9% of preterm group mothers had high school education. Majority of fathers in term group (47.6%) had university education, and 65.9% of fathers in preterm group had high school education. Mann-Whitney test results showed that term infants’ fathers had significantly higher levels of education compared to preterm fathers (P<0.001). Majority of both term and preterm mothers (84.1%) were housewives, and majority of fathers in term group (69.5%) and in preterm group (78%) were self-employed. 37.7% of subjects owned their home, and others lived in rented accommodations, service houses, or with their families. However, the difference between the two mothers and fathers groups in terms of occupation, income level, and housing was insignificant (Table 1).

**Table 1:** Demographic data of Parents of Preterm and term infants

Birth type (Demographic data)	Term (N=82)	Preterm (N=82)	Result
Mother’s age(mean ±SD)	28.2±4.5	27.6±6.2	P=0.53
Father’s age (mean ±SD)	32.8±4.8	31.7±6.2	P=0.23
Mother’s education / number / (Percentage)			P=0.88
Primary school	13(15.9)	16(19.5)	
High school	42(51.2)	36(43.9)	
University	27(32.9)	30(36.6)	
Father’s education / number / (Percentage)			**P<0.001
Primary school	17(20.7)	28(34.1)	
High school	26(31.7)	54(65.9)	
University	39(47.6)	-	
Mother’s job (housewife)/number (Percentage)	69(84.1)	69(84.1)	P=1
Father’s job (self-emPloyed)/number (Percentage)	57(69.5)	64(78)	P=0.21
Family’s income level / Rials/ number / (Percentage)			
0 - 4000000	12(14.5)	9(11)	
4000000 - 8000000	34(41.5)	19(23.2)	P=0.056
8000000 - 10000000	20(24. 4)	38(46.3)	
10000000 - more	16(19.5)	16(19.5)	
Mother’s marriage age/(mean ±SD)	20.6±3.4	20.2±3.6	P=0.46

*P<0.05, ** P <0.01

In both groups, most subjects were primiparous, and no significant difference was observed between the two groups in terms of type of delivery, number of deliveries, number of living children, number of male children, number of female children, or history of miscarriage, stillbirth, and neonate’s gender. According to the results obtained, significant differences were observed between two groups of infants requiring hospitalization (P<0.001) and neonatal intensive care unit (P<0.001) (Table 2).

**Table 2:** Obstetric characteristics of Parents of Preterm and term infants and characteristics of newborns

Birth type (Pregnancy information)	Term (N=82)	Preterm (N=82)	Result
First Pregnancy/number (Percentage)	37(45.1)	40(48.8)	P=0.71
Number of child /number (Percentage)			
1	39(47.6)	43(52.4)	P=0.87
2-5	43(52.4)	57(47.6)	
Abortion history/number (Percentage)	15(81.7)	22(62.8)	P=0.19
Stillbirth history/number (Percentage)	6(7.3)	11(13.4)	P=0.2
Natural childbirth/number (Percentage)	38(46.3)	47(57.3)	P=0.11
Wanted Pregnancy from the mother’s Point of view	64(78)	51(61.2)	**P=0.01
Wanted Pregnancy from the father’s Point of view	64(78)	47(57.3)	*P=0.03
Failure of contracePtive methods	10(12.2)	23(28)	**P=0.01
Son (s)/number (Percentage)	50(61)	45(54.9)	P=0.42
Need for hosPitalization/number (Percentage)	9(11)	48(58.5)	**P<0.001
HosPitalization at ICU/number (Percentage)	3(3.7)	43(52.4)	**P<0.001
Ability to Pay costs/number (Percentage)	80(97.6)	70(85.4)	**P=0.01
Gestational age/weeks/(mean ±SD)	35.4±1.02	39.1±1.04	**P=0.01
Weight/gr/(mean ±SD)	2563.9 ±348	3359.2±373.9	**P=0.01

* P<0.05,

** P <0.01

In terms of incidence of disorders like gestational diabetes, gestational hypertension, urinary tract infection, vaginal infection, bleeding or staining, oligo- and poly-hydroamnios, or incidence of other gestational diseases, the differences were insignificant.

Results indicate a significant difference in posttraumatic stress between mothers in two groups (P=0.03), and incidence of posttraumatic stress disorder in mothers of preterm infants was significantly higher than in mothers of term infants. However, according to Fisher’s Exact Test, this difference was insignificant between two groups’ fathers (Table 3).

**Table 3:** Absolute and relative frequency distribution of PTSD in Parents of both groups

Birth type (Mother’s PTSD)	Term Number (Percentage)	Preterm Number (Percentage)	χ^2^
Yes	1(1.2)	8(9.8)	
N/A	81(98.8)	74(90.2)	*P=0.03
Father’s PTSD			
Yes	2(2.4)	1(1.2)	P=1
N/A	80(97.6)	81(98.8)	

*P<0.05, ** P <0.01

Results show that, in terms of coping strategies with stress, there was a significant difference between the two groups of mothers (P<0.001) and the two groups of fathers (P<0.001) (Tables 4 and 5). A mutual effect was observed in mothers’ group between strategy adopted and type of birth (term, preterm) (P=0.006). This mutual effect was not found in the fathers’ group (P=0.33). Of mothers with posttraumatic stress disorder, 11.1% used anxiety, 44.4% emotion-oriented, and 44.4% used problem-oriented coping styles with stress. In fathers with posttraumatic stress, use of each of the coping styles was 33.3%.

**Table 4:** Absolute and relative frequency distribution of coping strategies in mothers of both groups

Birth type (Copping strategies)	Problem-oriented	emotion-oriented	anxiety
Term/mean(standard deviation)	42.28 (6.55)	47.4(6.51)	49.72 (8.29)
Preterm/mean(standard deviation)	47.52 (6.89)	50.59(7.93)	50.29(7.1)
Results (RM ANOVA)			**P<0.001
Mutual effect			**P=0.006

*P<0.05, ** P <0.01

**Table 5:** Absolute and relative frequency distribution of coping strategies in Fathers of both groups

Birth type (Copping strategies)	Problem-oriented	emotion-oriented	anxiety
Term/mean(standard deviation)	46.22 (7.23)	47.4 (6.51)	49 (8.31)
Preterm/mean(standard deviation)	48.35 (7.31)	50.59(7.93)	53.04 (7.69)
Results (RM ANOVA)			**P =0.001
Mutual effect			P=0.33

*P<0.05, ** P <0.01

The logistic regression model showed no significant correlations between posttraumatic stress in mothers and age, occupation, education, economic status, house statue, pregnancy times, history of infant hospitalization, history of infant admitted to neonatal intensive care unit, wanted pregnancy, and wanted infant’s gender. However, this model showed a significant correlation between posttraumatic stress and housing and coping strategies with stress in mothers (Table 6).

**Table 6:** Logistic regression model for PTSD of mothers

	OR	p-value	OR for 95% CI
Mother’s age	0.339	0.091	0.097-1.189
Mother’s occupation	0.82	0.106	0.004-1.701
Mother’s education	1.089	0.849	0.453-2.613
Economic statue	4.899	*0.034	1.124-21.385
House statue	0.121	0.060	0.013-1.097
Pregnancy times	0.724	0.538	0.259-2.024
Ability to Pay costs	0.000	0.998	0.000
Wanted Pregnancy from mother’s viewpoint	8.800	0. 84	0.749-103.384
Wanted infant’s gender from father’s view	1.509	0.781	0.083-27.602
Infant hospitalization	0.827	0.884	0.064-10.685
NICU hospitalization	0.773	0.362	0.064-10.685
Copping style	0.139	**0.006	0.34-0.573

*p<0.05, ** p <0.01.

Logistic regression model showed no correlation in fathers’ Posttraumatic stress disorder and age, occupation, education, house statue, history of infant hospitalization, history of infant admitted to sPecial care unit, wanted Pregnancy, wanted infant’s gender, or coping styles with stress (Table 7).

**Table 7:** Logistic regression model for PTSD of fathers

	OR	p-value	OR for 95% CI
Father’s age	3.095	0.219	0.511-18.757
Father’s occupation	3.711E7	0.997	0.000
Father’s education	1.486	0.450	0.531-4.161
House statue	2.294	0.581	0.120-43.785
Wanted Pregnancy from father’s viewpoint	11.693	0.116	0.547-250.092
Wanted infant’s gender from father’s viewpoint	2.847	0.558	0.086-93.909
infant hospitalization	1.754	0.804	0.021-149.318
NICU hospitalization	1.160	0.657	0.602-2.234
Copping style	1.930E7	0.995	0.000

*p<0.05, ** p <0.01.

## 4. Discussion

Few studies have examined psychological disorders in parents of premature infants in their early infancy, and studies have mostly considered psychological status of the mother and neglected the father. Moreover, coping strategies that can be highly effective in providing more appropriate coping styles for parents of premature infants and increase their efficacy and parenting quality have been overlooked in these studies. In addition, there are various conflicts in the relationship between coping strategies and outcomes of parents of premature infants in these studies.

According to the present study, mothers of preterm infants experience posttraumatic stress significantly more than mothers of term infants do. The results for posttraumatic stress in mothers in the present study are in line with those by Gambia et al. conducted in 2011 with the aim to compare levels of stress, anxiety, and depression in mothers of term and preterm infants. They found posttraumatic stress in mothers of premature infants was significantly higher than in mothers of term infants, but the differences between two studies were for questionnaire and time of collecting data, Gambina et al used MSP scale (Psychological Stress Measure), 3 days after delivery and before discharge from hospital, but in the present study data were collected 2 months after delivery. Also, Gray et al. conducted a study in Australia in 2012 with the aim to determine parental stress and psychological health in mothers of very immature infants compared to that in mothers of term infants, and concluded there was insignificant difference in the overall stress score between term and preterm groups, which disagrees with the results of present study. The difference between the two studies (Gray’s and the present study) was that we used the above questionnaires 2 months after birth to investigate psychological disorders in parents, but Gray et al. collected the questionnaires 4 months after birth. Besides, they used Parenting Stress Index- Short Form (PSI-SF) scale to investigate mothers’ stress, which is different from the posttraumatic stress disorder symptoms scale used in present study. Furthermore, Gray et al. only investigated psychological status of mothers, and ignored fathers’ mental state. Participants in Gray et al. study were mothers of infants born in their gestational age of 24-30 weeks that were admitted to special care unit. In the present study, mothers of infants born in their gestational age of 32-36 weeks participated as the preterm group, and only 52.4% of them were admitted to special care unit.

According to the results of present study, no difference was observed in posttraumatic stress in fathers in term and preterm groups. These results are in line with results of the study by Pierrehumbert et al. conducted in 2003 aiming to investigate effects of parents posttraumatic reactions on feeding and sleeping of their child, and also with results of the study by Kersting et al. conducted in 2004 aiming to compare posttraumatic stress symptoms in mother of term and preterm infants. According to Pierrehumbert results, parents of premature infants had more posttraumatic stress than parents of term infants. It was also observed that severity of prenatal risks increases the likelihood of incidence of posttraumatic stress in parents. According to results of this study, 67% of mothers showed symptoms of posttraumatic stress, and in the term group, this level was only 6%. Intensity of posttraumatic reactions in parents is a strong predictor of behavioral and sleeps problems of their child. Kersting et al. found that mothers of premature infants showed significantly more symptoms of posttraumatic stress disorder on days 1-3, 14, and months 6 and 14 after birth compared to mothers of term infants, and reduction in severity of these symptoms was observed 14 months after birth. The difference between the present study and Kersting et al study was in the timing of collecting data, and also their follow-up of mothers in terms of posttraumatic stress disorder.

In a study by Shaw et al. in 2009 aiming to investigate prevalence of posttraumatic stress disorder in parents of preterm or sick infants 4 months after birth and to find a relationship between acute stress symptoms immediately after birth and posttraumatic stress disorder, 32.22% of fathers and 9% of mothers were affected by posttraumatic stress disorder. Symptoms of acute stress significantly correlated with posttraumatic stress disorder and depression. Appearance of posttraumatic stress in fathers was more delayed compared to mothers, but on the fourth month, they were more exposed to risks than mothers. 54.4% of mothers showed complete symptoms of acute stress disorder. However, fathers did not show such symptoms. Differences between this study and current study were in methods and aim of study.

In a cross-sectional interventional study in 2005, Jotzo et al. investigated mothers of preterm infants admitted to special care unit (Jotzo & Poets, 2005). Mothers in the intervention group received a structured psychological intervention in the first few days after birth. All mothers could use psychological support if they needed. Control group mothers received no intervention, but could request counseling. When infant was discharged from hospital, mothers in both groups completed a questionnaire containing key outcome parameters (trauma symptoms, feelings when discharged, and sample and control variables. Results of this study showed no difference in demographic variables, family status, pregnancy, and birth in the two groups. At discharge, levels of traumatic response symptoms in intervention group mothers (25 mothers) was significantly lower compared to control group mothers (25 mothers). Thus, conclusion was drawn that intervention program for parents of preterm infants which was a mixture of early crisis intervention, psychological help when infant was admitted, and strong support at times of crisis reduced trauma symptoms associated with prematurity.

According to the present study, fathers of preterm and term infants showed insignificant differences in terms of posttraumatic stress disorder. Thus, in terms of stress, findings of the present study disagree with those in Sloan et al. study in 2008 titled “stress and coping in fathers of preterm infants”. This study aimed to investigate experience of fathers of preterm infants during their hospitalization. According to Sloan’s results, fathers reported moderate levels of stress, and considered their parents as the main source of social and emotional support and nurses and doctors as the main source of information support. Fathers were also inclined to use adaptive coping strategies with stress, and tried to re-shape the circumstances. The difference between the present study and Sloan’s was in the timing of collecting data and in tools used because data were collected when infants were admitted to special care unit, and Maslach scale was used to investigate stress, and structured interview for social support and coping strategies with stress.

According to results of the present study, significant differences were found in use of each of the 3 approaches: problem-oriented, emotion-oriented, and anxiety as dominant coping styles in parents of both term and preterm groups. A review of similar studies showed that Affleck and Tennen in 1991 investigated coping strategies of parents of preterm infants when their infant had been admitted to special care unit, and found that the most common strategies used by parents were moderate, investigative, and group supports. Greater use of escapist strategies was associated with lesser positive mood at discharge from hospital, while more use of minimalistic strategies was associated with higher positive mood.

Davis et al. in 2003 found no relationship between coping strategies and depression symptoms in mothers of very immature infants a month after birth. Reichman et al. in 2000 found that coping strategies of escape-avoidance had the strongest relationship with psychological distress of parents of very low birth weight infants.

## 5. Conclusion

Results of present study show that posttraumatic stress disorder in mothers of preterm group was significantly higher than that in mothers of term infants, and there was also a significant difference between parents in two groups in strategies adopted to cope with stress. Yet, it seems that, even after 2 months since birth, parents of preterm infants were more exposed to posttraumatic stress disorder than parents of term infants, and there is a need to adopt educational approaches to improve parents’ coping ability with immaturity of their infant. In future studies, fathers should also be considered more.

## References

[ref1] Affleck G, Tennen H (1991). The Effect of Newborn Intensive Care on Parents’ Pschological Well-Being. Children’s Health Care.

[ref2] Davis L, Edwards H, Mohay H, Wollin J (2003). The impact of very premature birth on the psychological health of mothers. Early human development.

[ref3] Foa E. B, Tolin D. F (2000). Comparison of the PTSD Symptom Scale–Interview Version and the clinician-administered PTSD Scale. Journal of Traumatic Stress.

[ref4] Gambina I, Soldera G, Benevento B, Trivellato P, Visentin S, Cavallin F, Zanardo V (2011). Postpartum psychosocial distress and late preterm delivery. Journal of Reproductive and Infant Psychology.

[ref5] Gray P. H, Edwards D. M, O’Callaghan M. J, Cuskelly M (2012). Parenting stress in mothers of preterm infants during early infancy. Early human development.

[ref6] Jahromi B. N, Salarian L, Shiravani Z (2011). Maternal risk factors and neonatal outcome of the admitted patients for preterm spontaneous uterine contractions. Iranian Red Crescent Medical Journal.

[ref7] Jotzo M, Poets C. F (2005). Helping parents cope with the trauma of premature birth: an evaluation of a trauma-preventive psychological intervention. Pediatrics.

[ref8] Kersting A, Dorsch M, Wesselmann U, Lüdorff K, Witthaut J, Ohrmann P, Arolt V (2004). Maternal posttraumatic stress response after the birth of a very low-birth-weight infant. Journal of psychosomatic research.

[ref9] Lima de Souza N, Pinheiro-Fernandes A. C (2010). Domestic maternal experience with preterm newborn children. Revista de Salud Pública.

[ref10] Melnyk B. M, Feinstein N. F, Alpert-Gillis L, Fairbanks E, Crean H. F, Sinkin R. A, Gross S. J (2006). Reducing premature infants’ length of stay and improving parents’ mental health outcomes with the Creating Opportunities for Parent Empowerment (COPE) neonatal intensive care unit program: a randomized, controlled trial. Pediatrics.

[ref11] Mirzamani S. A, Mohammadi M. R, Besharat M. A (2006). Application of the PTSD symptoms scale (PSS) for Iranian PTSD patients. Medical Journal of the Islamic Republic of Iran (MJIRI).

[ref12] Pierrehumbert B, Nicole A, Muller-Nix C, Forcada-Guex M, Ansermet F (2003). Parental post-traumatic reactions after premature birth: implications for sleeping and eating problems in the infant. Archives of Disease in Childhood-Fetal and Neonatal Edition.

[ref13] Reichman S. R, Feldman M, A Cate G, Robert M, Hendricks-Munoz K. D (2000). Stress appraisal and coping in mothers of NICU infants. Children’s Health Care.

[ref14] Shaban Z, Dolatian M, Shams J, Alavi-Majd H, Mahmoodi Z, Sajjadi H (2013). Post-Traumatic Stress Disorder (PTSD) Following Childbirth: Prevalence and Contributing Factors. Iran Red Cres Med J.

[ref15] Shah P. E, Clements M, Poehlmann J (2011). Maternal resolution of grief after preterm birth: implications for infant attachment security. Pediatrics.

[ref16] Shaw R. J, Bernard R. S, DeBlois T, Ikuta L. M, Ginzburg K, Koopman C (2009). The relationship between acute stress disorder and posttraumatic stress disorder in the neonatal intensive care unit. Psychosomatics.

[ref17] Shokri O, Taghilou S, Geravand F, Paeizi M, Moulaei M, Elahpour M, Akbari H (2008). Factor structure and psychometricks properties in Farsi version of the Coping Inventory with Stressful Situation (CISS). Advances in Cognitive Science.

[ref18] Sloan K, Rowe J, Jones L (2008). Stress and coping in fathers following the birth of a preterm infant. Journal of Neonatal Nursing.

[ref19] Subramaniam A, Abramovici A, Andrews W. W, Tita A. T (2012). Antimicrobials for preterm birth prevention: an overview. Infectious diseases in obstetrics and gynecology, 2012.

